# Effective practices of international volunteering for health: perspectives from partner organizations

**DOI:** 10.1186/s12992-018-0329-x

**Published:** 2018-01-24

**Authors:** Benjamin J. Lough, Rebecca Tiessen, Judith N. Lasker

**Affiliations:** 1 School of Social Work University of Illinois at Urbana-Champaign, 1010 W. Nevada St, Urbana, IL 61801 USA; 2 School of International Development and Global Studies, University of Ottawa, Ottawa, Canada; 3 Department of Sociology and Anthropology, Lehigh University, Bethlehem, USA; 4 Faculty of Humanities, University ofJohannesburg, Johannesburg, South Africa

**Keywords:** International volunteering, Quantitative, Training

## Abstract

**Background:**

The demand for international volunteer experiences to promote global health and nutrition is increasing and numerous studies have documented the experiences of the international volunteers who travel abroad; however, little is known about effective practices from the perspective of partner organizations. This study aims to understand how variables such as the skill-level of volunteers, the duration of service, cultural and language training, and other key variables affect partner organizations’ perceptions of volunteer effectiveness at promoting healthcare and nutrition.

**Method:**

This study used a cross-sectional design to survey a convenience sample of 288 volunteer partner organizations located in 68 countries. Principle components analyses and manual coding of cases resulted in a categorization of five generalized types of international volunteering. Differences among these types were compared by the duration of service, skill-level of volunteers, and the volunteers’ perceived fit with organizational needs. In addition, a multivariate ordinary least square regression tested associations between nine different characteristics/activities and the volunteers’ perceived effectiveness at promoting healthcare and nutrition.

**Results:**

Partner organizations viewed highly-skilled volunteers serving for a short-term abroad as the most effective at promoting healthcare and nutrition in their organizations, followed by slightly less-skilled long-term volunteers. The greatest amount of variance in perceived effectiveness was volunteers’ ability to speak the local language, followed by their skill level and the duration of service abroad. In addition, volunteer training in community development principles and practices was significantly related to perceived effectiveness.

**Conclusion:**

The perceptions of effective healthcare promotion identified by partner organizations suggest that program and volunteer characteristics need to be carefully considered when deciding on methods of volunteer preparation and engagement. By better integrating evidence-based practices into their program models, international volunteer cooperation organizations can greatly strengthen their efforts to promote more effective and valuable healthcare and nutrition interventions in partner communities.

## Background

Many of today’s global development and relief organizations depend on volunteers for training, capacity building, and service provision. Each year, governments, inter-governmental organizations, educational and faith-based organizations, NGOs and other stakeholders spend millions of dollars on promoting, marketing, and administering international volunteer programs [1, 2]. While there are diverse models of international volunteering (South-North, South-South), this study is concerned with the dominant model of North-South international development volunteering –or the large and diverse practices of international volunteers living in the Global North volunteering in Global South communities with the aim of development assistance programming [3, 4].^1^ Within this sector, demand for international volunteering from pre-health and graduate health professions is particularly popular, and medical student and resident training programs with global health electives have risen significantly since the early 2000s [5, 6]. The United Nations Volunteers program found that volunteers specializing in healthcare were in higher demand by communities in the Global South than volunteers specializing in many other professions [7].

A growing body of scholarly literature and non-academic reports have examined the motivations, roles and impacts of international volunteers [8–11]. Much of this research has focused on the experiences of the volunteers, and occasionally on the organizations that sponsor their trips [11]. However, little is known about what constitutes desirable and effective practices of international volunteering from the perspective of partner organizations [12]. A key assumption underlying this study is that volunteer partner organizations (VPOs)—the local organizations that host volunteers from abroad—are better able to articulate factors that contribute to effective practices than are volunteers or sending organizations who act on decisions in the absence of local perspectives.

This study brings in new and original insights to the study of international volunteering. While much of the scholarship focuses on the experiences and perspectives of the international volunteers, this study offers innovative insights from the perspectives of the participating organizations. Specifically, this study focuses on understanding how different types of health-related volunteering are perceived by organizations that host these volunteers. Their assessments, often undervalued and ignored, are essential to improving the opportunities, and minimizing the challenges and limitations of international health volunteering [13–15]. In most cases, these VPOs provide significant benefits to volunteers, and are local experts on what is needed in their organizations and communities to best achieve shared goals [16]. The following section begins this discussion with a review of literature on international health volunteerism. It highlights several weaknesses and strengths of international volunteers, including an overview of important ethical considerations for international volunteering in the health sector.

## Research review of medical volunteerism practices

A growing scholarly critique of volunteer activities focuses on the unethical practice of medicine with vulnerable patients by untrained students [17–21]. These studies have included interviews with medical personnel [22–24], medical and non-medical staff who work with volunteers [11], community members and patients [25–27], or a combination of all of the above [28, 29]. The results of such studies, despite disparate methods, locales, and programs, are remarkably consistent and valuable in identifying ways in which health-related volunteer programs should be improved. Volunteer-host partner organizations, community members, and staff in the Global South have pointed to some key challenges with managing volunteers and have raised questions about their effectiveness in meeting development practice and outcomes [12, 13]. For example, VPOs raise concerns about volunteers’ lack of cultural understanding, displaying attitudes of superiority, disrespecting local customs and practices, and imposing their own methods and opinions in ways that are inappropriate to the practice environment [11, 22, 24]. They also express a desire for greater continuity of care and better communication with volunteers, both in terms of language and in clarity of purpose. Some raise the possibility of foreign physicians competing with, or even replacing, locally trained professionals [28].

Research with experienced medical officers in sub-Saharan Africa in 2005 highlighted additional concerns about international health volunteering—reporting an overall larger number of negative experiences with international health volunteers than positive experiences [30]. Among the challenges identified by these locally-based health experts, volunteers were perceived as too “junior, inexperienced and ill prepared to work in low-income countries”, more likely to experience “difficulties with cultural and language barriers, and with differences in norms and values, resulting from insufficient cultural sensitivity and awareness”; a general lack of understanding about local health practices and challenges; and limited skill sets and training to work in these new environments. Other concerns raised in this study included an “undervaluing [of] local staff knowledge” and a perceived unwillingness “to support the public health system, resulting from a lack of understanding of their role and lack of communication on their terms of reference, job description and mutual expectations” [30].

Along with these criticisms, however, staff, patients and community members in the studies described above also report satisfaction with the experience of hosting volunteers; they appreciate the concern shown by visitors for underserved people, the “extra hands” they provide in severely understaffed situations, and the medical services and supplies they typically bring with them [2]. Interviews with 55 staff members in health VPOs in four countries reported very positive perceptions about the contributions of volunteers hosted by their programs [2]. Likewise, Laleman et al.’s [30] study investigating international health volunteering in sub-Saharan Africa reported that locally-based health experts believed international volunteers were particularly helpful for their hard work, motivation, adaptability, dedication, high capacity to innovate, and their ability to teach specialized skills [30]. Other research on healthcare volunteering with VPOs in Indonesia and India found that short-term skilled medical volunteers were effective at disseminating knowledge and expertise, with 97% of respondents valuing the transfer of medical technical skills provided by volunteers [14]. The Catholic Health Association, which carried out a survey of 49 host country VPOs in 14 countries, found that VPOs were happy to receive volunteers who demonstrated a willingness to learn from their hosts, had knowledge of the local language and culture, shared their technical skills, and provided training for local staff [31].

Commentators on the mixed benefits and drawbacks of international volunteer trips recommend a number of changes, with many pointing to strategies to improve them. Also, organizations have developed guidelines for promoting more effective and responsible volunteer missions in global health. After reviewing 27 guidelines for health-related volunteer trips, Lasker et al. identified five commonly mentioned themes [15]. More than half of these guidelines mentioned the need for a collaborative partnership with a host organization, preparation of volunteers, evaluation of impact, cultural competence/language, and training of host medical staff. Better recruitment of volunteers and better matching of volunteer skills to host needs were also mentioned, though by a smaller number of guidelines. Other research has confirmed that host organizations often view cultural humility and teamwork as far more important than clinical or technical skills alone [32].

### Collaborative and mutually-beneficial partnerships

Consistent with these studies, VPOs often request a far more significant role in the partnership beyond providing logistical support. Beyond having their voices heard, VPO staff report wanting equal weight and voice in decision-making about which volunteers to accept, and about the specific activities volunteers will engage in. They ask that their feedback be taken seriously by volunteers and volunteer organizations [23–25, 27, 31]. One study that surveyed VPO staff and community members in the Dominican Republic reported a “misalignment of the desired and actual skill sets of volunteers; duplicate and uncoordinated volunteer efforts; and the perpetuation of stereotypes suggesting that international volunteers possess superior knowledge or skills” [33]. Lasker identified “mutuality”, or the opportunity for all people involved to be learning and developing skills, as one of the most important principles for effective volunteering [2]. However, this goal is not often achieved in practice. Smaldino, Lasker, & Myser analyzed how perceived power differentials between VPOs and international visitors can make mutuality hard to achieve, even when this principle is considered a key goal of the programs [34]. As Kumwenda [23] concluded following his interviews with host staff at sites in three African countries:

*The challenge to both students and their sending institutions is to progress towards giving something proportionate back in return for the learning experiences received. There is clearly room to improve electives from the hosts’ perspective, but individually host institutions lack the opportunity or ability to achieve change* (p. 623).

Some of the important ethical considerations emerging from highly skilled volunteers engaged in international health electives point to the inequality perpetuated when mutuality is lacking. Wealthy medical students from high-income countries have used under-funded and under resourced clinics as teaching spaces where they can gain experience that benefits the volunteers with little positive impact on the host community [5, 35]. The one-way benefits of international health training for Global North students and interns is compounded by the dependency produced when resource-poor clinics come to rely on the steady flow of affluent medical students and healthcare professionals, the resources they bring, the services they provide and the financial investments they make in the communities. Such dependency on external resources are susceptible to gaps in volunteer visits, expectations of good will and donations accumulated by the volunteer, among other unknowns of unbalanced relationships [35].

### Duration of volunteer service

The duration of volunteer service has emerged as one important consideration tied to VPO satisfaction. With the exception of some medical students volunteering abroad, international healthcare volunteers are typically highly skilled professionals and, consequently, can only serve for a short-term (often less than 2 weeks), though hosting organizations prefer 3 week or longer [11]. In Laleman et al.’s [30] study, although short-term volunteers with specialized medical training were highly valued by host organizations, those who were able to stay for longer periods of time (i.e. around 2 years) were preferred [30]. Likewise, although VPOs reporting on their experiences with short-term medical volunteers from Singapore highly valued the volunteers’ expertise, 62% of the VPOs believed that the trainings, which were often limited to a few days, were too short to learn many new skills [14]. Overall, while there is some lack of consensus about the overall weight of duration on service effectiveness—often depending on the type of VPO and the skill-level of the local partners, longer durations (i.e. 1 year or longer) are typically preferred.

### Volunteer training, cultural and language competencies

Volunteer training is also often discussed as an important practice associated with effective practice. Volunteers who participate in promoting healthcare rarely have specific training in culturally-relevant medical procedures, general cultural practices, and international development principles [30, 36]. In Laleman et al.’s [30] study, volunteers who had undergone language training, and who had expertise in tropical medicine, epidemiology and/or health service organization were preferred over volunteers who lacked such training [30].

In connection with volunteering training, a large and growing number of less-skilled or unskilled volunteers, often self-described ‘pre-medical’ students, are going abroad to participate in health-related programs. For many of these volunteers, their experience revolves around the prospect of learning with few restrictions. Many pre-health professions students are told they need “clinical experience” in their applications for professional programs, something that is hard to gain in their home countries where restrictions are much greater [37, 38]. Because unskilled volunteers are sometimes presented with opportunities to perform medical procedures for which they have no training, it often results in significant ethical challenges for both volunteers and partner organizations [12, 34].

Although previous research has examined a number of variables and principles associated with effective practices, much of this research is based on case studies and qualitative inquiry. Studies using empirical quantitative data to assess the perspective of VPOs that host volunteers are rare [12]. The current study is the most extensive survey of VPOs’ perspectives to date. It goes beyond prior research by including views of VPOs from multiple countries to better assess and understand their perceptions of volunteering effectiveness. Given the wide variety of program models, which of these options do VPOs that host volunteers prefer? This study aims to answer these questions by assessing factors most highly associated with the perceived effectiveness of healthcare delivery by international volunteers.

## Methods

Two major players in the growing international volunteering industry are international volunteer cooperation organizations (IVCOs) based in countries that send volunteers overseas and volunteer partner organizations (VPOs) in communities in low- and middle-income countries that host the volunteers. The target population for this study included VPOs located in the Global South. There are tens of thousands of such organizations around the world, with no listing that would allow contacting a random sample of VPOs. However, many IVCOs that partner with VPOs are members of networks that coordinate international volunteering. Researchers created a formal collaboration with six key international volunteer service networks (IVSNs) that worked with their members IVCO to distribute surveys to partner VPOs around the world.

In addition to providing access to VPOs with an interest in improving volunteer practices, the research collaboration with IVSNs and IVCOs helped to ensure the active engagement and consultation of non-academics in the survey and research design stages of the study and to ensure that research goals were mutually beneficial. Thus, non-academic partners played an active role identifying survey participants in the partner countries, in implementing the research protocol, and in helping to interpret results.

### Recruitment of survey participants

Collaborating IVSNs sent a brief information packet to all IVCOs within their networks, describing the research and requesting their participation and consent to collaborate in the research project. As a result of these requests, the researchers established collaborative partnerships with a total of 46 IVCOs. IVCOs were located in the US (37%), Canada (13%), Germany (13%), Australia (5%), Spain (5%), and the UK (4%), Korea (3%), and nine other high-income countries. These IVCOs were asked to select a sample of VPOs in the countries where they worked that might be willing to participate in the survey. Two criteria were placed on the selection of VPOs, which included: (1) the VPOs should each have a minimum of 1 year working history with the IVCOs, and (2) they should have hosted a minimum of three international volunteers.

### Survey instrument

Researchers developed a survey to assess how various practices of international volunteering affect diverse outcomes -- including but not limited to the promotion of healthcare and nutrition. VPOs were asked to identify their organization’s most important priority areas among a list of 20 pre-identified categories. “Healthcare promotion / disease prevention / maternal or child health” was one of these main categories. VPOs were also asked to estimate what percentage of international volunteers had served for various lengths of time in their organization. Respondents were presented with six response options to measure duration ranging from “less than 1 week” to “1 year or more”. Respondents were further asked to rate how many international volunteers working with their organization had particular traits (including being highly skilled, having competencies that fit with organizational needs, etc.). These responses options were presented on a 5-point Likert scale ranging from “none” to “all”. Finally, respondents were asked to rate how effective international volunteers were at promoting healthcare and nutrition in their organization. These responses options were presented on a 5-point Likert scale ranging from “very poor” to “excellent”.

### Survey administration

The survey was administered online to all contacts identified by the 46 collaborating IVCOs. These surveys were translated into three languages (English, French, and Spanish). Collaborating IVCOs were given two choices for the administration of surveys to their partners. As one option, the IVCOs could send the researchers contact details for their VPOs. As a second option, the IVCOs could contact their partners directly with an anonymous link to the survey. In the first case, one follow-up email was sent. In the second case, no follow-up email was sent, as the researchers had no method for tracking the rate of response or participation. In all cases, participation was completely voluntary.

All surveys were taken by an administrator of the participating VPOs—typically the executive officer. Among organizations contacted directly by the researchers, 1,130 VPOs received the survey and 239 responded (22%). The response rate among VPOs contacted by the IVCOs is unknown as partner IVCOs were not able to articulate how many VPOs were contacted; however, 81 VPOs among this group responded. A few surveys were dropped from the analysis due to incomplete responses. In total, the analysis includes 288 survey responses from VPOs operating across 68 low- and middle-income countries. Many of these VPOs were located in Southeast Asia, with more than 15% of VPOs located in either India or Indonesia. Around 23% of partner organizations were located in the African continent. Participating VPOs reported hosting most of their volunteers from South Korea (44%), the USA (35%), Germany (26%), France (15%), the UK (14%), Canada (12%), Japan (12%), Switzerland (11%), and Australia (10%).

### Data analysis

In order to assess differences across the diverse characteristics of volunteer programs, we carried out an initial principle components analysis (PCA). Variables included in the PCA included the duration of volunteer service; volunteers’ education, skills, and competencies; group placement status; minimum age of volunteers accepted by VPOs; and the resources expended and/or received by VPOs to host volunteers (if any). PCA yielded three broad categories (individual long-term volunteers, highly skilled and older short-term volunteers, and medium-term volunteers). However, several variables failed to load well on any of these three components, with other variables loading on multiple components. Overall, a viable solution could not be attained from PCA alone based on overlapping constructs (for e.g. short-term volunteers were alternately perceived as both highly skilled and unskilled). This led the researchers to code each case response manually, based on their knowledge of the sending programs combined with a manual inspection of survey responses on the duration of service, volunteer age, and the skill- and educational-level of volunteers.

Manual coding of case responses resulted in five broad categories of volunteers represented in the survey responses: less-skilled long-term, less-skilled short-term, semi-skilled medium-term, skilled short-term, and skilled long-term volunteers. These categories were heuristically determined rather than by formulaic computation of skill-level and duration of service. Nonetheless, some categories do follow general “types” of international volunteering. *Skilled short-term volunteers* have significant skills, training and experience and usually serve for less than 8 weeks because they often maintain concurrent employment [39–41]. *Skilled long-term volunteers* typically live and work in low-income communities for one year or more, and are usually required to hold a college degree as a minimum educational requirement [42–44]. They were the most common form of volunteers in our sample and are often referred to as “development volunteers” because the long-term skilled volunteering model has a long-standing historical precedence tied to Western development theory and practice [45]. *Less-skilled short-term* volunteering has also been referred to as volunteer tourism or “voluntourism” in the literature, and is often performed by young people with few marketable skills [46, 47]. *Unskilled long-term* and *semi-skilled medium-term* volunteers are not common categories in written literature or scholarly examination. Although these forms did not fit any of the three main forms of international volunteering often discussed in scholarship, they were evident in the data and represent the variety and flexibility of volunteering options for people interested in serving abroad. Table 1 illustrate differences in these five categories by duration of service, skill-level of volunteers, and their perceived fit with organizational needs. This five-category typology of volunteers was used to illustrate how categorical differences impacted the level of volunteers’ perceived effectiveness at promoting healthcare and nutrition.

**Table 1:** Descriptive characteristics of VPOs by category (*n* = 286 VPOs)

	Number	Average days	Healthcare a priority area	Highly Skilled	Competency Fit with Org Needs
Less-skilled short-term volunteers	31	44	32.3%	13.3%	41.9%
Less-skilled long-term volunteers	53	416	26.4%	8.5%	40.7%
Semi-skilled medium-term volunteers	36	169	33.3%	42.4%	83.3%
Skilled short-term volunteers	39	35	23.1%	97.4%	92.1%
Skilled long-term volunteers	127	447	20.5%	85.7%	88.1%

To assess bivariate differences among VPOs that listed healthcare and disease prevention as a key priority (*n* = 71), the researchers completed a series of bivariate or chi-square tests, as well as Analysis of Variance (ANOVA) tests, followed by pairwise comparisons using a Tukey post hoc test to determine statistically significant differences. Because of the complicated nature of reporting Tukey tests for 5-category responses, bivariate statistics were not reported in tables but are reported in the text.

Researchers also ran a multivariate OLS linear regression to analyze how differences in nine volunteer characteristics and activities are associated with the level of volunteers’ perceived effective at promoting healthcare and nutrition. The multivariate analysis was used to better control for the multiple influences of duration and skills on perceived effectiveness, and to assess additional variables previously associated with volunteer effectiveness. Nine variables included in the multivariate model include the computed number of days volunteers served (originally a 6-category response); partners’ perceptions about the degree of volunteers who are highly skilled, culturally sensitive, from a higher-income country, highly motivated, and speak the local language (5-category responses); and binary responses about whether volunteers received the following types of training before or during service with their organization: community development training, cross-cultural training, and language training. Prior to entering variables in the regression model, univariate analyses were completed to verify that assumptions of regression were met. Likewise, bivariate correlations and distributions between variables included in the model were all well within acceptable ranges.

## Results

### Characteristics of VPOs

The majority (53%) of participating VPOs were non-governmental organizations, followed by government or quasi-governmental organizations (35%). The remaining 12% included educational, for-profit, and faith-based organizations. On average, the participating organizations had been receiving volunteers for around 10 years. The priority area of “healthcare promotion / disease prevention / maternal or child health” was the third highest area prioritized by the VPOs (26%). This priority focus was preceded only by primary and secondary education (35%) and youth development and youth services (27%).

### Differences in health prioritization among VPOs

VPOs that listed healthcare as a key priority were significantly different from other VPOs in several ways. First, they were more likely to describe themselves as NGOs (35%) rather than government (14%) organizations (*χ*^*2*^ = 12.5, *df* = 1, *p* < .001). Also, all faith-based organizations (*n* = 6) listed health as one of their major priorities. VPOs reporting a priority in healthcare were also more likely to give priority to economic development (*p* < .001), environmental sustainability (*p* < .001), humanitarian relief (*p* < .001), and primary and secondary education (*p* < .001) than VPOs that did not list health as a priority.

There was no difference among VPOs in the ideal length of time international volunteers stayed with the organization (*p* = .20; 71% of all the VPOs considered 6 months or more to be ideal). Likewise, although descriptive statistics indicate some difference (see Table 1), healthcare was rated as equally important among VPOs that hosted different types of volunteers (*χ*^*2*^ = 3.74, *df* = 4, *p* = .44).

With bivariate analysis, the perceived skill level of volunteers was positively correlated with VPO’s perception of effectiveness at promoting healthcare and nutrition in their organizations (*p* < .001). However, there were some important differences between categories. Although VPOs rated high-skilled short-term volunteers as more effective (good or excellent = 64.3%) than the other four categories of volunteers; they only rated 47.2% of skilled long-term volunteers as good or excellent in health promotion (descriptive differences between categories are presented in Table 2). Thus, a percentage of the perceived skills of long-term volunteers were in areas other than the promotion of healthcare and nutrition.

**Table 2:** How effective are international volunteers at promoting healthcare and nutrition in your organization?

	Very Poor	Poor	Average	Good	Excellent
Less-skilled long-term volunteers (*n* = 31)	3.2%	16.1%	41.9%	29.0%	9.7%
Less-skilled short-term vols. (*n* = 23)	4.3%	26.1%	26.1%	17.4%	26.1%
Semi-skilled medium-term vols. (*n* = 28)	0.0%	7.1%	32.1%	57.1%	3.6%
Skilled long-term volunteers (*n* = 72)	6.9%	9.7%	36.1%	31.9%	15.3%
Skilled short-term volunteers (*n* = 28)	0.0%	10.7%	25.0%	35.7%	28.6%
Total (*n* = 182)	3.8%	12.6%	33.5%	34.1%	15.9%

As evidenced in the multivariate analysis, the variable explaining the greatest amount of variance in perceived effectiveness was a volunteers’ ability to speak the local language (*t* = 3.06, *β* = .235, *p* < .01), followed by volunteers’ higher skill level (*t* = 2.67, *β* = .211, *p* < .01). (See Table 3). The duration of service was the next most influential variable, with perceived effectiveness of volunteers decreasing with each additional day volunteers serve (*t* = 2.63, *β* = .201, *p* < .01). Finally, the training of volunteer in community development principles and practices was significantly related to effectiveness (*t* = 2.46, *β* = .177, *p* < .05). The perceived cultural sensitivity of volunteers, and language training provided to volunteers were also marginally associated with volunteers’ perceived effectiveness but only with 90% confidence (*p* < .10).

**Table 3:** Level of volunteers’ perceived effectiveness at promoting healthcare and nutrition (*n* = 168)

	B	SE	*β*	*t*	*p*
(Constant)	2.937	.422		6.97	.000
Average number of days volunteers serve	−.001	.000	−.201	−2.63	.009
Are highly skilled	.198	.074	.211	2.67	.008
Are culturally sensitive	.105	.060	.132	1.76	.080
Are from a higher-income country	.108	.071	.121	1.52	.130
Are highly motivated	−.033	.098	−.029	−.33	.740
Receive community development training	.399	.162	.177	2.46	.015
Receive cross-cultural training	.117	.159	.055	.74	.462
Receive language training	−.284	.156	−.135	−1.82	.071
Speak the local language	.193	.063	.235	3.06	.003

## Discussion and implications

### Study limitations

Before attempting to interpret these findings, a number of limitations should be acknowledged. First, although the sample of VPOs may be the largest included in research to date, they cannot fully represent the range of VPOs that host international volunteers, particularly in health-related programs. The fact that the largest percentage of VPOs’ host volunteers come from South Korea reveals a significant sampling bias. An IVCO in South Korea that specializes in sending skilled long-term volunteers abroad was more determined than others at encouraging their partners to respond. If truly proportional, the sample should have far more representation from VPOs in the US, Canada, Australia, UK, and Germany. It is unclear how this might have affected results, compared to having predominantly white volunteers coming primarily from countries with more significant colonial and post-colonial influences.

As another limitation in the sample, the IVCOs approached to participate in this research are all members of IVSNs that prioritize community development over other alternative objectives; they therefore represent a particular niche of organizations that are concerned with development effectiveness and are often valued partners in projects with other transnational development organizations. This makes them different in (often unknown) ways from the range of organizations that host health-related volunteers. This bias may also help explain why faith-based VPOs are only 2.1% of the total sample, surely a much smaller proportion than in volunteer programs generally (see for example [48]).

Additionally, an unknown proportion of the international volunteer industry does not work in close collaboration with specific VPOs. In a survey of 177 U.S.-based sponsor organizations, almost half indicated that they do not always have a partner in the host country [2]. Sponsor organizations in the Global North sometimes work directly with individual pastors, doctors or political leaders. Some simply show up in a rural area and set up a clinic or other type of program. Thus, the VPO model is only a part of the story; indeed, the partnership with hosting organizations may be far more effective compared to other less partnership-focused volunteer programs [49, 50].

Although these data represent the perspectives of VPOs as a heretofore under-represented voice, we recognize that VPOs also have biases, interests and preferences that may influence their evaluation of volunteer effectiveness. Because this study does not draw correlations between the subjective views of VPOs and more objective client or patient outcomes, we cannot verify whether the volunteers are truly effective at promoting healthcare and nutrition in the partner organizations.

Given the structural limitations of accessing the desired skills and expertise of highly trained healthcare professionals, it is possible that VPOs have completed these assessments of effectiveness based on the best perceived option available, rather than on their ideal model of international support that may be implemented in a more perfect world. Thus, findings may reflect their lived perceptions of “the best we can get” rather than perceptions of truly effective practice.

Data analysis also presents a few key limitations. First, the categorization of volunteer programs into five generalized types is inevitably reductionist; some of the variability contained within cases will be lost during analysis. Second, the heuristic determination of cases into one of these five categories, was nearly as much art as science. Because the respondents specified the names of sending IVCOs, researchers were able to reasonably categorize the models based on the actual characteristics of each organization. However, the categorization of each case was less objective in situations where VPOs hosted volunteers from more than one IVCO. As a result, the boundaries embodying these categorizes are somewhat diffuse.

### Preferences of volunteer partner organizations

Although short-term skilled volunteers were viewed as comparatively more effective at promoting healthcare than long-term volunteers, this finding is somewhat conflated. The typical skilled long-term volunteer model recruits young people who recently received a college or university degree and are taking a “gap year” before starting their formal careers [30, 45]. In contrast, studies of skilled short-term medical volunteers typically describe these volunteers as working professionals with full-time jobs in their home countries [14, 51, 52]. Data from this study also indicate that skilled short-term volunteers are perceived as being 2 years older on average, and with a higher skill set in comparison with their long-term counterparts. Therefore, the effects of duration of service should not be divorced from the potential mediating effects of the volunteers’ skill-level.

Another reason for the perceived preference for shorter-term volunteers could be the toll that volunteers take on the time of local medical staff [53, 54]. It takes staff time and organizational resources to host volunteers—often diverting staff responsibilities from service delivery to managing volunteers. Findings from prior studies indicate that professional organizations (e.g. hospitals and clinics) may prefer to host short-term volunteers because it reduces the diversion of staff time from service delivery [14, 55, 56].

As this study indicates, less-skilled healthcare volunteers are not viewed by VPOs as particularly effective, even when they are willing to volunteer in the organization for a year or longer. Although a longer term of service is often used as a key variable in scholarly discussions about the effectiveness of healthcare volunteers [51], the skill level of volunteers accounts for more variance in a multivariate analysis than duration—even accounting for differences in duration. This is one important revelation coming out of this study, one that refutes conclusions from other studies (see [10]) that a longer duration of service is correlated with VPO satisfaction-- at least in the field of health-care provision.

One question that remains unresolved is: can short-term volunteer programs that provide highly qualified and well-prepared healthcare volunteers offer valuable, and possibly preferred models, of international volunteering? The study did not ask whether VPOs would welcome or prefer highly skilled short-term volunteers to stay for longer periods of time. While short demonstrations of volunteer services may fill a significant need, they may remain less-than-ideal options for volunteer receiving organizations if shorter time frames limit community health education and client care that could be provided if volunteers had language proficiency and longer-term capacity-building and follow-up [14, 56, 57].

As indicated in the multivariate analysis, volunteers’ ability to speak the local language explained the greatest amount of variability predicting the volunteers’ perceived effectiveness—explaining more variance than even their perceived level of skill. Language facility of the international volunteers speaks to the importance of ease of communication between the international and local healthcare staff and also the ability of the volunteers to communicate with the patients with whom they are interacting [14, 58]. Thus, the nature of practicing healthcare in communities where they must interact with diverse groups of people may require greater proficiency in the local/national language than volunteer work that is limited to interactions with office-based, and other highly educated staff members that may speak a common tongue [59].

Diverse types of training that healthcare volunteers receive before or during their service appeared to be mixed and inconsistent. Receiving training in “community development” principles before or during their service had a significant overall impact on perceived effectiveness. This could be explained, in part, by the nature of the community development training received by international volunteers, which often pairs volunteers with local professionals and mentors who understand the culture and context of healthcare service [14, 42]. Training based in the community also has implications for the degree of mutuality inherent in the volunteering partnerships, as partners are often involved in these training exercises [60]. Training in community development helps set a context for the contributions and limitations that volunteers can expect to contribute to the capacity of VPOs and the surrounding community. Indeed, the application of medical knowledge and theory to local contexts has been identified as a very important component of effective international healthcare volunteering [14].

The lack of significant association between cross-cultural and language training and perceived effectiveness is somewhat consistent with previous research [32]. This finding can also be explained, in part, by the variance in language training as explained in the multivariate model (i.e. by the volunteers’ ability to speak the local language). Likewise, VPOs perceptions of volunteers’ cultural sensitivity, in combination with other variables in the model, explained much of the variability around the effects of cross-cultural training. Given the low probability value associated with cultural sensitivity, it is likely that the power of this model to explain the effects of cultural sensitivity on perceived effectiveness may not have been high enough to reveal differences from organizational-level units of analysis.

## Conclusion

These data provide empirical evidence to inform our understanding about effective practices for health volunteering. Of critical importance, these data are based on the often undervalued and under-researched views and perceptions of partner organizations that host international volunteers. Taking these views under careful consideration can inform many of the perceived risks and challenges evident in previous research. Although some challenges will always exist with international volunteering, these data indicate that challenges to the effectiveness of healthcare volunteers are less prevalent when volunteers are highly skilled and have competencies that fit the needs of VPOs. These data also present findings that link the perceived effectiveness of international volunteers with a shorter duration of service, volunteers’ capacity to speak the local language, and the importance of receiving basic training in community development principles and practices.

Finding a match between volunteer competencies and host-country needs should take high priority for IVCOs and VPOs alike. The urgent need for highly-skilled healthcare volunteers to assist with life-saving support and other forms of capacity-building may overshadow other values and desires on the part of the VPOs. These findings, while pointing to a preference for skilled short-term healthcare volunteers, do not offer a full exploration of the most ideal form of international health volunteering. An important next step for future research would be to look at concrete health outcomes associated with different types of international volunteers. This would help assess whether subjective preferences for certain types of volunteering on the part of VPOs objectively correlate with clinical outcomes in practice.

In sum, the perceptions of effective healthcare practice identified by partner organizations suggest that the important issues of service duration, volunteer skill-level, language capacity, and training in the local community need to be carefully considered when deciding on the practices of volunteer preparation and engagement in international healthcare. By better integrating these and other evidence-based practices into their program models, international volunteer cooperation organizations can greatly strengthen their efforts to promote global health—delivering more effective and valuable healthcare interventions in partner communities.

## Footnotes

1 The North–South categorization reflects a global socio-economic and political divide. The North covers mostly higher-income countries in the northern hemisphere (in addition to Australia and New Zealand). The South covers lower-income countries generally located in the southern hemisphere.
